# Same-day discharge after percutaneous closure of persistent foramen ovale using intracardiac echocardiography and the Gore Septal Occluder

**DOI:** 10.3389/fcvm.2024.1408543

**Published:** 2024-06-26

**Authors:** Kristoffer Steiner, Gunnar Sjöberg, Anna Damlin, Magnus Settergren, Dinos Verouhis

**Affiliations:** ^1^ Department of Women’s and Children’s Health, Karolinska Institutet, Stockholm, Sweden; ^2^ Department of Paediatric Cardiology, Astrid Lindgren Children’s Hospital, Karolinska University Hospital, Stockholm, Sweden; ^3^ Department of Cardiology, Karolinska University Hospital, Stockholm, Sweden; ^4^ Unit of Cardiology, Department of Medicine, Karolinska Institutet, Stockholm, Sweden

**Keywords:** PFO closure, same-day discharge, intracardiac echocardiography, stroke prevention, Gore Septal Occluder (GSO), vascular closure device (VCD)

## Abstract

**Aim:**

Periprocedural and postinterventional care of patients undergoing closure of patent foramen ovale (PFO) varies significantly across care providers. Same-day discharge (SDD) after transcatheter interventions is an evolving concept. This study aimed to assess the same-day discharge rate and incidence of complications in patients undergoing PFO closure with intracardiac echocardiography (ICE) using the Gore®Cardioform Septal Occluder (GSO) device. The secondary aim was to analyse the efficacy of femoral vein closure with Perclose ProGlide.

**Methods:**

Patients who underwent PFO closure with the GSO device at a university hospital in Stockholm, Sweden, were retrospectively included between March 1, 2017, and June 30, 2020, all with cryptogenic stroke as the indication for the procedure. All patients underwent PFO closure with conscious sedation and local anaesthesia. The indication for all patients was a cryptogenic stroke. Periprocedural imaging was performed using ICE and fluoroscopy in all patients. Patient characteristics and periprocedural data were collected from patient charts. Patients were kept on bed rest for 4–6 h post-intervention. Transthoracic echocardiography and clinical examination, including groin status, were performed before discharge. No clinical routine follow-up was performed the day following the intervention. Clinical follow-up was done by phone call two weeks after the procedure, and echocardiographic follow-up was done after 12 months. Data were analysed using linear and logistic regression models.

**Results:**

In total, 262 patients were included, of which 246 (94%) had SDD. 166 patients (63%) received the ProGlide™ system for femoral vein access closure. Post-procedural arrhythmias occurred in 17 (6%) patients, and vascular complications in 9 patients (3%). The overall closure rate at follow-up was 98.5%. 25 out of 264 patients (9.5%) had to be readmitted within the first eight weeks after PFO closure, 16 due to atrial fibrillation warranting electric cardioversion, one due to an arteriovenous fistula that was operated, four due to chest pain/pain at the access site, and four patients developed fever. There was no difference in SDD among patients who received ProGlide™ vs. patients who did not receive ProGlide™.

**Conclusion:**

SDD appears safe after transcatheter PFO closure with the GSO device with high procedural success rates. Low rates of complications and readmissions make the intervention suitable for this patient-friendly and cost-effective concept.

## Introduction

The first clinical report of a paradoxical embolisation across a persistent foramen ovale (PFO) leading to systemic embolisation through the fossa Sylvii was written by Cohnheim in 1877 (1), as well as its importance in other clinical settings such as Platypnea–orthodeoxia syndrome and decompression sickness (2–4).

PFO closure can be performed surgically using direct sutures or patch closures (5), often requiring extended hospital stays, or as a catheter-based intervention with dedicated PFO closure devices, which has become the primary treatment option during the last two decades (5). Antiplatelet therapy has previously been the recommended therapy for patients with cryptogenic stroke (6, 7). Several randomised controlled trials and meta-analyses have reported a lower risk for recurrent ischemic stroke after PFO closure compared to medical therapy (8–12), leading to an increased number of PFO closures in the last five years.

Developments within several areas of interventional cardiology have aided in making procedures less invasive, allowing for early discharge from the hospital. Vascular closure devices (VCD) provide reliable access site control and allow for rapid mobilisation after procedures. These improvements have allowed for an increasing possibility of same-day discharge (SDD) after catheter-based interventions (13–18). An extensive review and summary discussing the feasibility of SDD after numerous transcatheter procedures was published by Asbeutah et al. (18).

This study aimed to assess the same-day discharge feasibility and, safety, and incidence of complications in patients undergoing PFO closure with intracardiac echocardiography (ICE) using the Gore®Cardioform Septal Occluder (GSO) device. The secondary aim was to analyse the efficacy of femoral vein access closure with Perclose ProGlide™.

## Methods

### Ethical approval

An ethical permit from the Swedish Ethical Review Authority has been approved with the registration number: (2022-02255-01).

### Study design and study participants

This retrospective, single-centre, register-based cohort study included patients from the Swedish registry of congenital heart disease (SWEDCON) (19). The SWEDCON register is a National Quality Registry frequently used for research projects due to its high quality and good follow-up data. It consists of subregisters for adult patients with congenital heart defects, children with congenital heart defects, paediatric cardiothoracic surgery, foetal register and a register for cardiomyopathies. Patients who underwent transcatheter PFO closure between March 1st 2017, and June 30th 2020, at the Karolinska University Hospital, Stockholm, were included for analysis. Cryptogenic stroke was the indication for all PFO closures in this study.

In total, 262 patients (98 females, 164 males) were included, of which 261 received a GSO device. Mean age at intervention was 46.3 years (SD ± 10.4), mean BMI 25.5 (SD ± 4.2). Cardiovascular risk factors such as arterial hypertension were noted in 32 patients (12.2%), Diabetes mellitus in 3 patients (1.1%), hypercholesterolemia in 29 patients (11%) and smoking in 11 patients (4%), see Table 1.

**Table 1:** Patient characteristics.

	All patients
Number of patients	262
Age	46.3 (±10.4)
Females Males	98 (37) 164 (63)
Weight	79 (±15.3)
BMI	25.5 (±4.2)
Hypertension	32 (12.2%)
Diabetes mellitus	3 (1.1%)
Hypercholesterolemia	29 (11%)
Smoking	11 (4%)

Data is presented in parentheses as mean and standard deviation (SD). Sex distribution and cardiovascular risk factors (hypertension, diabetes, hypercholesterolemia and smoking) are presented as a number (%). BMI is presented in mean, kg/m2 (SD).

BMI, body mass index.

### Preprocedural evaluation

All patients underwent TTE and TOE with administration of agitated saline as a diagnostic evaluation after cryptogenic stroke to verify the PFO diagnosis. Before the intervention, all patients were evaluated at a multidisciplinary team conference by an interventional cardiologist, neurologist, clinical physiologist and neuroradiologist, where the decision to proceed with percutaneous PFO closure was made.

### PFO closure procedure

All interventions were performed in a monoplane catheterisation laboratory using conscious sedation and local anaesthesia. Femoral venous access was obtained in all patients using a micropuncture technique with ultrasound guidance to minimise puncture-related complications. Two ipsilateral punctures were performed to allow access for the PFO-closure device and ICE catheter for intraprocedural imaging. An 11 Fr introducer was inserted into the femoral vein for device delivery. An ICE catheter (Abbot View Flex, Abbott Vascular, CA, USA) was introduced through a 9 Fr introducer and placed in the right atrium. Right heart catheterisation and the PFO closure were performed according to IFU.

After the procedure, patients were observed in the cardiology ward or daycare facility for 4–6 h, including a bedside TTE, to confirm the position of the device *in situ* and the absence of pericardial effusion. Patients were kept on strict bed rest for the first hour, followed by mobilisation. The antiplatelet regimen used for most patients was lifelong acetylsalicylic acid (160 mg once daily) combined with clopidogrel (75 mg once daily) for six months. Depending on the patient's comorbidity and preexisting therapy, anticoagulation (e.g., apixaban or coumadin) was continued with life-long acetylsalicylic acid.

### Access site closure

**Figure 1:**
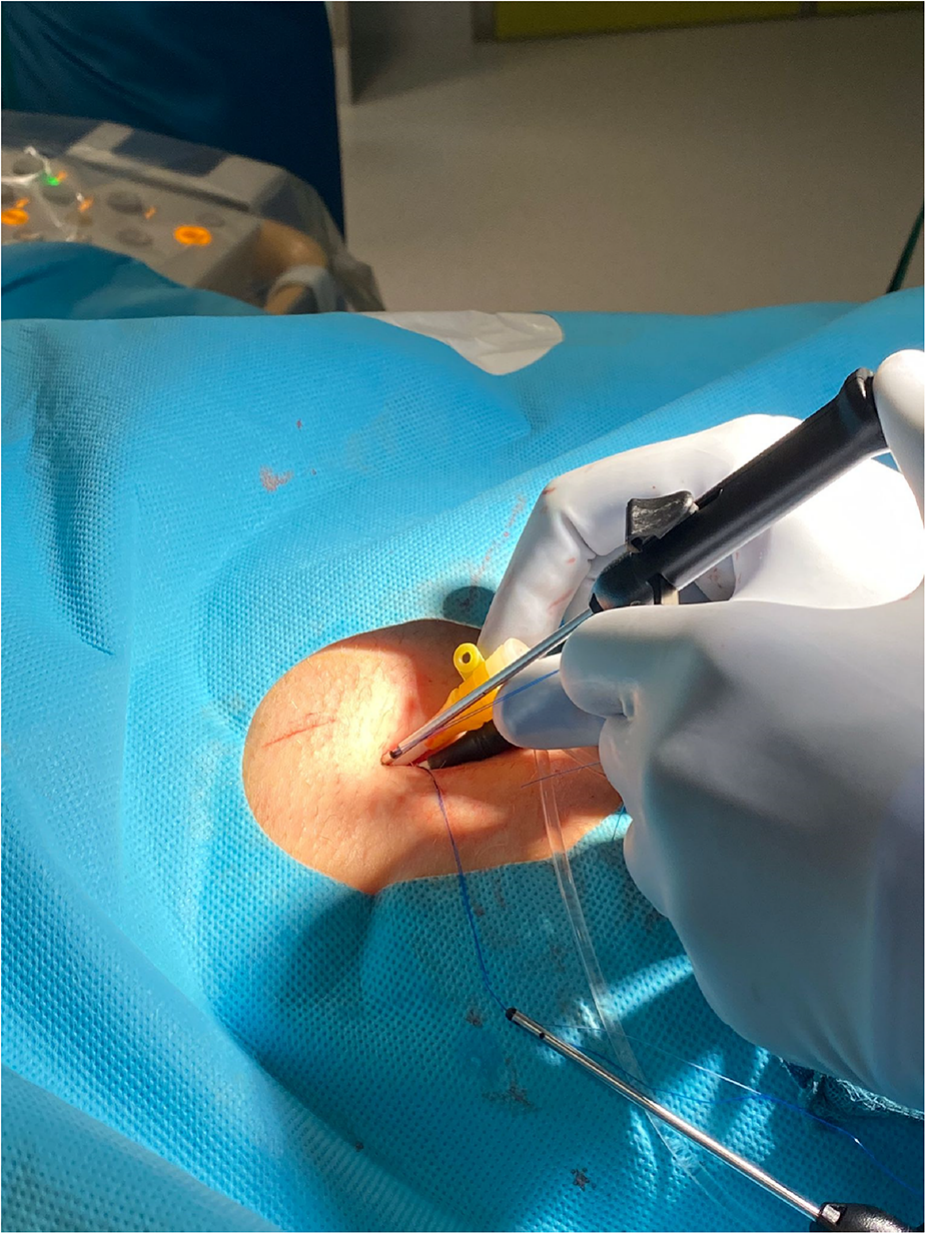
Access site closure with perclose proglide. Courtesy Magnus Settergren.

Access site closure was performed at the sole discretion of the treating interventionist, using preclosure with either the Perclose ProGlide™ system (Abbott Vascular, Santa Clara, California, USA) for each femoral venous puncture site or a figure-of-eight suture see Figure 1.

### Data collection

Baseline and periprocedural characteristics were retrospectively collected from patient charts. Bleeding or puncture-related complications were analysed following the Bleeding Academic Research Consortium Definition for Bleeding (BARC) criteria (20).

### Data analysis

Categorical variables were analysed using logistic regression models and described as proportions (percentages). Continuous variables were analysed using linear regression models and expressed as mean values with standard deviations (SDs). In all analyses, *P*-values < 0.05 were considered statistically significant. All data obtained were registered in Microsoft Excel® (Microsoft, Redmond, Washington, USA). Analyses were performed using STATA software (version 16.1 Stata Corp., College Station, Texas, USA).

## Results

SDD was achieved in 246/262 patients (94%). In patients with VCD, SDD was achieved in 159/166 patients (96%) and in 87/96 patients (91%) with figure-of-eight suture (*p* = 0.1).

The PFO device used most often was the 25 mm device in 164 patients (62%), followed by the 30 mm device in 93 patients (35%) and the 20 mm device in 4 patients (3%).

Vascular complications were seen in 9 patients (3%), 4 of these in patients with VCD and 5 in Non-VCD patients (*p* = 0.24).

Procedure-related atrial arrhythmias occurred in 17 (6%) patients, atrial fibrillation in 16 patients, and atrial flutter in one patient.

Periprocedural characteristics are summarised in Table 2. Complications depending on the femoral access closure approach are shown in Table 3. A comparison of SDD rates between patients receiving a VCD and patients with no VCD is shown in Table 4.

**Table 2:** Periprocedural characteristics.

Patients	262 (100)
SDD	246 (93.9%)
GSO device 20mm	4 (3%)
GSO device 25mm	164 (62%)
GSO device 30mm	93 (35%)
Perclose ProGlide™ device	166 (63%)
Closure rate	258 (98.5)
Procedure time	35 (± 15)
Fluoroscopy time	8 (± 6.2)
Follow-up time	12.7 (10.1)

Data are presented as number and percentage in parenthesis (patients, SDD, device), mean time in minutes ± standard deviation (procedure time, fluoroscopy time), or mean time in months ± standard deviation (follow-up).

GSO, gore cardioform septal occluder; SDD, same-day discharge; SD, standard deviation.

**Table 3:** Comparison of complications depending on femoral access closure approach.

	All patients	VCD	Non-VCD	*P*-value*
Number of patients	262 (100)	166 (63)	96 (37)	
Vascular complication	9 (3)	4 (2)	5 (5)	0.24
Any complication	38 (15)	24 (14)	14 (15)	0.98

* *P*-values when comparing VCD and non-VCD patients using linear regression model for age comparison and logistic regression models for all other parameters. All values are described in parentheses as numbers and percentages. Complications: including arrhythmias, pain at access site, fever.

**Table 4:** Comparison of same-day discharge rates between patients receiving a VCD and patients with no VCD.

	All patients	VCD	Non-VCD	*P*-value*
Patients SDD, *n* (% of patients)	262 (100) 246 (94)	166 (100) 159 (96)	96 (100) 87 (91)	0.10

* *P*-values when comparing VCD and non-VCD patients using logistic regression models. All values are described in parentheses as numbers and percentages. SDD, same-day discharge.

### Same-day discharge

SDD was achieved in 246 (93.9%) patients. Among the patients with delayed discharge (i.e., no SDD), the following complications were registered: vascular complications (*n* = 3), late procedure starting time (*n* = 4), pain in the groin (*n* = 2), epigastric pain (*n* = 1), drainage of pericardial effusion (*n* = 2), post-procedural atrial fibrillation (*n* = 1), post-procedural desaturation (*n* = 1), post-procedural pulmonary complications (*n* = 1), and personal reasons (*n* = 1). The reasons for post-procedural readmissions and emergency room (ER) visits were atrial fibrillation (arrhythmia *n* = 16, 6.1%), fever (*n* = 4, 1.5%) or chest pain/pain at the access site (*n* = 4, 1.5%). One patient was operated on due to an AV fistula five weeks post-PFO closure.

### Complications

The Perclose ProGlide™ system was used for access site closure in 166 patients, five (3%) of whom suffered vascular complications. Three had minor venous bleeding after application of the Perclose ProGlide™ and were treated with additional figure-of-eight sutures in the catheterisation laboratory. Technical failure at deployment of the ProGlide™ system occurred in one patient, and the access site was closed with a figure-of-eight suture instead. One patient was readmitted to the hospital due to vascular complications and underwent surgery due to an arteriovenous (AV) fistula five weeks post-PFO closure. No other patient had to be readmitted due to vascular complications. There were two type 3b (cardiac tamponade) bleeding complications and eight type 1 bleeding complications, according to BARC (20). There were no differences in complications between patients who received the ProGlide™ device and those who did not (Table 3).

## Discussion

The findings of this study support that SDD after PFO closure can be safely achieved in a majority of patients. Early, or SDD, is evolving for numerous transcatheter interventions such as aortic valve implantations, coronary intervention mitral valve procedures, and PFO closure (13–17). PFO closure is performed as right heart catheterisation from the venous side, with considerably smaller delivery catheters than transcatheter aortic valve implantations or mitral valve procedures, making the intervention suitable for early discharge.

Three previous studies have evaluated SDD after PFO closure using different PFO devices: Gore Helex Occluder and the Amplatzer Occluder (21); Amplatzer PFO occluder (22), Gore Cardioform Septal Occluder and Amplatzer PFO Occluder (23). The success rate of PFO closures in this study was higher than in previous studies. The higher use of GSO devices in this study could have contributed to the higher success rates, as the GSO's different design with Polytetrafluoroethylene optimises for complete closure.

### The choice of anaesthesiology method can also influence the postoperative course

All patients in this study underwent PFO closure with conscious sedation and general anaesthesia, which can facilitate early discharge. Postoperative nausea and vomiting (PONV) is a common adverse effect of general anaesthesia affecting up to 80% of patients (24) and may prolong hospital stay. The primary risk factor for PONV is the use of volatile anaesthetics. Performing the intervention with conscious sedation and local anaesthesia facilitates SDD by reducing the rate of PONV. An argued advantage of general anaesthesia with intubation is the possibility of performing TOE, but it can prolong procedural duration. Other options are TOE without general anaesthesia and/or with only a brief TOE exam at the end of the procedure (18).

At our institution, ICE and fluoroscopy are the standard protocols for imaging of the atrial septum, enabling procedures without TOE and general anaesthesia.

The perioperative imaging guided by ICE rather than TOE reduces the necessity for general anaesthesia and lessens the risk of associated complications (25, 26). Hence, the use of ICE during all the procedures in this study possibly contributed to the low rate of complications and high rate of SDD. This is supported by Blusztein et al., presenting that ICE and avoiding general anaesthesia were highly and significantly associated with SDD (23). They reported SDD in 382/554 patients (68.9%), with ICE used in 503/554 patients (90.7%), ICE and TOE were used in another three patients, and the remaining patients were treated using fluoroscopy or TOE, concluding that SDD was more common in patients with ICE.

In a retrospective study by Barker et al., SDD was achieved in 456/467 patients (97.6%) (22). Similar to our study, patients underwent PFO closure using local anaesthesia and conscious sedation. Periprocedural imaging in the study by Barker et al. was done using ICE and fluoroscopy in 86/467 patients (18.4%); the remaining 381 patients underwent closure with fluoroscopy alone.

High success rates of SDD rates were seen in the study by Barker et al. (22), in our study and in the subgroup of patients with local anaesthesia in the study by Bluzstein et al (23), suggesting that local anaesthesia is associated with SDD.

An extensive overview discussing the advantages and disadvantages of ICE for PFO closure has been published by Egidy Assenza (27). Another approach was described by Achim et al., using fluoroscopy as imaging during the procedure with a short TOE control before device release to assess device positioning and stability. This approach lead to significantly shorter procedural length in the fluoroscopy-guided group with no difference in procedural complications, including death, major bleeding, device dislodgement, stroke or clinically relevant peripheral embolisation between the two groups (*p* = .99) (28).

Applying a VCD such as the ProGlide™ Perclose system can further increase the possibility and safety of early discharge. Sekhar et al. reported the device to be safe and effective for femoral artery closure after heart catheterisation before early discharge (29). In our study, there was no difference overall in SDD rate between the patients who received the ProGlide™ device and those who did not receive the ProGlide™ device.

Late vascular problems were rarely seen in our study. One patient (0.38%) was diagnosed with an AV fistula one week after the procedure and underwent vascular surgery five weeks after the initial procedure.

Arrhythmia was the most common complication seen after the PFO closures in this study. The most encountered arrhythmias after PFO closure are known to be atrial tachycardias such as supraventricular tachycardia (SVT), atrial fibrillation (AFib) and atrial flutter (Af) (REF). Readmission rates are mainly driven by atrial fibrillation (AFib) requiring treatment, as demonstrated in this study and supported by Abrahamyan et al. (30). There is a low risk of clinically significant atrioventricular disturbances such as complete atrioventricular block (AVB) requiring pacemaker implantation (30). The risk of SVT requiring medical therapy or electrical cardioversion was reported to be around 1.5% in a large study by Szkutnik (31); late AVB requiring pacemaker was seen in 2/739 patients (0.27%) after 4.3 years respectively 1.5 years after Amplatzer device implantation. This reflects the results in our study, where acute onset AF delayed hospital discharge in one patient and caused ER visits in another sixteen patients.

### Limitations

This study is mainly limited by the retrospective and observational design, which is susceptible to confounding. There was no randomisation to either SDD or VCD, which complicates the interpretation of the result.

## Conclusions

This retrospective study demonstrates the safety and feasibility of SDD in patients undergoing PFO closure for cryptogenic stroke with the GSO device. SDD after PFO closure can lead to healthcare savings and better utilisation of healthcare resources.

## Data Availability

The raw data supporting the conclusions of this article will be made available by the authors, without undue reservation.
